# Redefining Hepatocellular Carcinoma Staging Systems Based on the Bile Duct Invasion Status: A Multicenter Study

**DOI:** 10.3389/fonc.2021.673285

**Published:** 2021-10-14

**Authors:** Qizhen Huang, Yufeng Chen, Kongying Lin, Chuandong Sun, Shuguo Zheng, Jinhong Chen, Yifan Wang, Yanming Zhou, Weiping Zhou, Jingfeng Liu, Yongyi Zeng

**Affiliations:** ^1^ Department of Radiation Oncology, Mengchao Hepatobiliary Hospital of Fujian Medical University, Fuzhou, China; ^2^ Department of Hepatopancreatobiliary Surgery, Zhangzhou Affiliated Hospital of Fujian Medical University, Zhangzhou, China; ^3^ Department of Hepatopancreatobiliary Surgery, Mengchao Hepatobiliary Hospital of Fujian Medical University, Fuzhou, China; ^4^ Department of Hepatobiliary and Pancreatic Surgery, Affiliated Hospital of Qingdao University, Qingdao, China; ^5^ Institute of Hepatobiliary Surgery, Southwest Hospital, Third Military Medical University, Chongqing, China; ^6^ Department of General Surgery, Huashan Hospital, Fudan University, Shanghai, China; ^7^ Department of General Surgery, Sir Run Run Shaw Hospital, School of Medicine, Zhejiang University, Hangzhou, China; ^8^ Department of Hepato-Biliary-Pancreato-Vascular Surgery, First Affiliated Hospital of Xiamen University, Xiamen, China; ^9^ The Third Department of Hepatic Surgery, Eastern Hepatobiliary Surgery Hospital, Second Military Medical University, Shanghai, China; ^10^ Department of Hepatopancreatobiliary Surgery, First Affiliated Hospital of Fujian Medical University, Fuzhou, China

**Keywords:** hepatocellular carcinoma, bile duct invasion, staging system, survival, prognosis

## Abstract

**Background and Aims:**

The prognostic value of bile duct invasion (BDI) remains controversial. We aimed to investigate the prognostic value of BDI and the stage of BDI in different staging systems.

**Methods:**

Patients with hepatocellular carcinoma (HCC) from nine hepatobiliary medical centers who underwent R0 resection were included. Overall survival (OS) was assessed using the Kaplan–Meier method and tested using the log-rank test. The prognostic effect of BDI was analyzed using univariate and multivariate Cox proportional hazard regression analyses. The predictive performance of these models was evaluated using the concordance index and time-dependent receiver operating characteristic curve (tdAUC).

**Results:**

Of 1021 patients with HCC, 177 had BDI. OS was worse in the HCC with BDI group than in the HCC without BDI group (p<0.001); multivariate analysis identified BDI as an independent risk factor for OS. After adjustment for interference of confounding factors using the Cox proportional hazard regression model, HCC with BDI and without macrovascular invasion was classified as Barcelona Clinic Liver Cancer (BCLC) B, eighth edition American Joint Committee on Cancer (AJCC) IIIA, and China Liver Cancer (CNLC) IIb, respectively, whereas HCC with BDI and macrovascular was classified as BCLC C, AJCC IIIB, and CNLC IIIA, respectively. C-indexes and tdAUCs of the adjusted staging systems were superior to those of the corresponding current staging systems.

**Conclusion:**

We constructed adjusted staging systems with the BDI status, improved their predictive performance and facilitate clinical use.

## Introduction

Hepatocellular carcinoma (HCC) is the sixth common cancer and fourth cancer-specific cause of death globally (1). HCC is prone to invading the vessel system and forming a tumor thrombus during its development (2, 3). When HCC invades the bile duct wall and grows in the bile duct cavity, it is called bile duct invasion (BDI). The clinical incidence of BDI ranges from 0.45% to 12.9% (4–8) and appears to have increased in the last decade (8).

Previous studies have shown that the prognosis of HCC patients with BDI is not significantly different from that of those without BDI (4–6, 9–11). However, in recent years, many studies have come to the opposite conclusion and regard BDI as a risk factor for prognosis (12–18). Current clinical staging systems such as the Barcelona Clinic Liver Cancer (BCLC) staging system (19), eighth edition American Joint Committee on Cancer (8^th^ AJCC) staging system (20), and China Liver Cancer (CNLC) staging system (21) are widely used in clinical settings. These staging systems are composed of clinical indicators including general status, liver function status, tumor size, tumor number, vascular invasion, and extrahepatic metastasis. All the aforementioned indicators are significant risk factors for prognosis. Therefore, these staging systems have great clinical significance and guiding value for prognosis evaluation and treatment selection in HCC cases. However, BDI was not included in all the HCC staging systems.

The purpose of this study was to explore the survival differences between HCC with and without BDI to determine the prognostic value of BDI and to adjust current staging systems according to the BDI status.

## Patients and Methods

### Ethics Statement

This study was conducted according to the Declaration of Helsinki and the Ethical Guidelines for Clinical Studies and was approved by the institutional research ethics committee of the Mengchao Hepatobiliary Hospital of Fujian Medical University (approval number: 2020_077_01).

### Study Population

HCC patients with microscopic or macroscopic BDI admitted to one of the nine Chinese hepatobiliary medical centers (the Mengchao Hepatobiliary Hospital of Fujian Medical University, Eastern Hepatobiliary Surgery Hospital of Second Military Medical University, Zhangzhou Affiliated Hospital of Fujian Medical University, Affiliated Hospital of Qingdao University, Southwest Hospital of Army Medical University, Huashan Hospital of Fudan University, First Affiliated Hospital of Fujian Medical University, Sir RunRun Shaw Hospital of Zhejiang University School of Medicine, and First Affiliated Hospital of Xiamen University) between March 1, 2007 and March 1, 2018 were included. HCC patients without BDI were from the Mengchao Hepatobiliary Hospital of Fujian Medical University and Eastern Hepatobiliary Surgery Hospital of Second Military Medical University during the same period.

The inclusion criteria were as follows: 1) both HCC and BDI were histopathologically confirmed, 2) tumors were treated with R0 resection, and 3) complete clinical data and postoperative follow-up records. The exclusion criteria were as follows: 1) recurrent or metastatic HCC, 2) combined HCC-intrahepatic cholangiocarcinoma, 3) other accompanying cancers, and 4) clinical data or survival data are missing. R0 resection was defined as the removal of all macroscopic tumors with a microscopically negative margin. The decision of treatment depends on the discussion of the multidisciplinary team in each center, whether perform surgical resection mainly considering the liver function, residual liver volume, tumor related factors such as if complicated with portal vein main trunk tumor thrombus or vena cava tumor thrombus or distant metastasis, and whether tumor and tumor thrombus can be completely removed.

### Postoperative Follow-up

All patients were regularly followed up after discharge from the hospital. Follow-up visits were scheduled once every 2–3 months in the first 2 years, once every 6 months from 2 to 5 years, and once every year after 5 years. Follow-up examinations were conducted using laboratory tests [serum alpha-fetoprotein (AFP), liver function, and complete blood count], abdominal ultrasonography, and/or contrast-enhanced computed tomography magnetic resonance imaging. Overall survival (OS) was defined as the time of resection to the date of either death or the latest follow-up. Data including baseline and clinical characteristics and follow-up information were extracted and censored on September 31, 2020.

### Patients’ Clinicopathological Characteristics

Clinicopathological characteristics included age, sex, underlying liver diseases, cirrhosis, the number of tumors, maximum tumor size, presence of satellites and tumor differentiation, microvascular invasion (MVI), major vascular invasion, macrovascular invasion (MaVI), preoperative serum gamma-glutamyltransferase (GGT), alkaline phosphatase (ALP), total bilirubin (TBil), AFP, Child–Pugh grade, MELD (model for end-stage liver disease) score, BCLC stage, AJCC stage, and CNLC stage. Underlying liver diseases were categorized as viral liver disease (hepatitis B virus or hepatitis C virus), non-alcoholic fatty liver disease, alcoholic liver disease, and no underlying liver disease. Cirrhosis was confirmed histopathologically or *via* clinical diagnosis. PS (performance status) score refer to ECOG (Eastern Cooperative Oncology Group, ECOG) PS score. Tumor differentiation was classified according to the Edmonson–Steiner grade. MVI was vascular invasion of small vessels only identifiable histologically. Major vascular invasion was defined as invasion of the branches of the main portal vein (right or left portal vein, excluding the sectoral and segmental branches), one or more of the three hepatic veins (right, middle, or left), or the main branches of the proper hepatic artery (right or left hepatic artery) (20). Major vascular invasion was used in AJCC stage. MaVI was defined as vascular invasion of large vessels detectable radiologically or macroscopically. Major liver resection included 3 or more segments. Preoperative serum indicators selected in the study were the result of the most recent test within 15 days prior to surgery. Preoperative treatments included transarterial chemoembolization (TACE), hepatic arterial infusion chemotherapy (HAIC), and external radiotherapy. Postoperative adjuvant treatments included TACE, HAIC, systemic chemotherapy, and targeted agents. The patients who were not suitable for direct operation because of high bilirubin or liver dysfunction or other reasons received biliary drainage or supportive treatment (such as improve liver function, correction of anemia and hypoalbuminemia and management of complications) before operation. Definitions of the BCLC stage, AJCC stage, and CNLC stage were referenced from relevant clinical guidelines.

### Statistical Analysis

Continuous variables are reported as medians with interquartile ranges and were compared using Student’s t-test or the Mann–Whitney test. Categorical data, presented as frequencies (%), were compared using the chi-square test or Fisher’s exact test. Kaplan–Meier survival curves were used to assess OS. Univariate and multivariate analyses were performed using the Cox proportional hazard regression model, and a backward stepwise selection method was used to identify independent prognostic factors and adjust for confounding factors. The Harrell’s concordance index (C-index) (22) and time-dependent areas under the receiver operating characteristic curve (tdAUC) (23) were used to evaluate the predictive performance of each staging system. All statistical tests were two-tailed, and P<0.05 was considered significant. Statistical analyses were performed using IBM SPSS Statistics software (version 24.0; IBM Corp., Armonk, NY) and R version 3.6.1 (http://www.r-project.org/); the R packages of “readxl,” “table1,” “rms,” “survminer,” “survival,” “ggplot2,” “CsChange,” and “timeROC” were used.

## Results

### Baseline Characteristics

Of 1021 patients with HCC who were included, flowchart was shown in **Supplementary Figure 1**. 177 had BDI (BDI+) and 844 did not have BDI (BDI-). Compared with patients without BDI, those with BDI had higher PS scores, higher Child–Pugh grade, higher MELD score, higher TBil levels, higher GGT levels, higher ALP levels, more single tumors, lower differentiation grades, more complete capsules, more satellite nodules, a higher incidence of MVI and MaVI, received more extensive resection and were more likely to receive postoperative adjuvant treatment (all P<0.05). Patients’ baseline characteristics are listed in **Table 1**.

**Table 1:** Clinicopathological characteristics of patients with comparison between HCC without BDI and HCC with BDI.

Characteristics	All	Without BDI	BDI	P-value
	(n = 1021)	(n = 844)	(n = 177)	
**Age**				
Median [IQR]	51.0 [45.0, 59.0]	51.0 [44.0, 59.0]	53.0 [46.0, 59.0]	0.199
**Sex**				
Female	154 (15.1%)	125 (14.8%)	29 (16.4%)	0.677
Male	867 (84.9%)	719 (85.2%)	148 (83.6%)	
**Underlying liver disease**				
No	196 (19.2%)	162 (19.2%)	34 (19.2%)	0.935
virus	796 (78.0%)	657 (77.8%)	139 (78.5%)	
NASH	24 (2.4%)	21 (2.5%)	3 (1.7%)	
ALD	5 (0.5%)	4 (0.5%)	1 (0.6%)	
**Cirrhosis**				
No	359 (35.2%)	300 (35.5%)	59 (33.3%)	0.636
Yes	662 (64.8%)	544 (64.5%)	118 (66.7%)	
**PS**				
0	883 (86.5%)	745 (88.3%)	138 (78.0%)	<0.001
1	111 (10.9%)	81 (9.6%)	30 (16.9%)	
2	27 (2.6%)	18 (2.1%)	9 (5.1%)	
**Child-Pugh grade**				
A	926 (90.7%)	798 (94.5%)	128 (72.3%)	<0.001
B	95 (9.3%)	46 (5.5%)	49 (27.7%)	
**MELD score**				
Median [IQR]	3.11 [1.44, 4.81]	2.86 [1.36, 4.32]	6.18 [2.72, 8.83]	<0.001
**TBil(umol/L)**				
Median [IQR]	14.0 [10.8, 19.2]	13.4 [10.2, 17.4]	32.1 [15.5, 74.8]	<0.001
**ALP(U/L)**				
Median [IQR]	85.0 [66.0, 116]	80.0 [64.0, 100]	153 [97.0, 228]	<0.001
**GGT(U/L)**				
Median [IQR]	73.0 [38.0, 147]	61.0 [34.0, 116]	238 [132, 435]	<0.001
**AFP (ng/mL)**				
Median [IQR]	41.0 [6.30, 954]	50.5 [6.65, 994]	20.0 [4.60, 697]	0.197
**Tumor number**				
Single	830 (81.3%)	675 (80.0%)	155 (87.6%)	0.025
Multiple	191 (18.7%)	169 (20.0%)	22 (12.4%)	
**Tumor size(cm)**				
≤5	485 (47.5%)	397 (47.0%)	88 (49.7%)	0.571
>5	536 (52.5%)	447 (53.0%)	89 (50.3%)	
**Grade**				
I/II	124 (12.1%)	92 (10.9%)	32 (18.1%)	0.011
III/IV	897 (87.9%)	752 (89.1%)	145 (81.9%)	
**Capsule**				
No	896 (87.8%)	757 (89.7%)	139 (78.5%)	<0.001
Yes	125 (12.2%)	87 (10.3%)	38 (21.5%)	
**Satellite nodules**				
No	774 (75.8%)	658 (78.0%)	116 (65.5%)	<0.001
Yes	247 (24.2%)	186 (22.0%)	61 (34.5%)	
**MVI**				
No	609 (59.6%)	554 (65.6%)	55 (31.1%)	<0.001
Yes	412 (40.4%)	290 (34.4%)	122 (68.9%)	
**Major vascular invasion**				
No	934 (91.5%)	781 (92.5%)	153 (86.4%)	0.013
Yes	87 (8.5%)	63 (7.5%)	24 (13.6%)	
**MaVI**				
No	880 (86.2%)	738 (87.4%)	142 (80.2%)	0.016
Yes	141 (13.8%)	106 (12.6%)	35 (19.8%)	
**Number of resected segments**				
One	402 (39.4%)	350 (41.5%)	52 (29.4%)	<0.001
Two	376 (36.8%)	332 (39.3%)	44 (24.9%)	
Three	150 (14.7%)	93 (11.0%)	57 (32.2%)	
Four and more	93 (9.1%)	69 (8.2%)	24 (13.6%)	
**Major/minor liver resection**				
Minor	778 (76.2%)	682 (80.8%)	96 (54.2%)	<0.001
Major	243 (23.8%)	162 (19.2%)	81 (45.8%)	
**Preoperative treatment**				
No	1001 (98.0%)	831 (98.5%)	170 (96.0%)	0.07
Yes	20 (2.0%)	13 (1.5%)	7 (4.0%)	
**Postoperative adjuvant treatment**				
No	582 (57.0%)	513 (60.8%)	69 (39.0%)	<0.001
Yes	439 (43.0%)	331 (39.2%)	108 (61.0%)	
**BCLC stage**				
0	56 (5.5%)	50 (5.9%)	6 (3.4%)	0.017
A	631 (61.8%)	531 (62.9%)	100 (56.5%)	
B	100 (9.8%)	85 (10.1%)	15 (8.5%)	
C	234 (22.9%)	178 (21.1%)	56 (31.6%)	
**8^th^ AJCC stage**				
IA	59 (5.8%)	51 (6.0%)	8 (4.5%)	<0.001
IB	445 (43.6%)	404 (47.9%)	41 (23.2%)	
II	336 (32.9%)	240 (28.4%)	96 (54.2%)	
IIIA	94 (9.2%)	86 (10.2%)	8 (4.5%)	
IIIB	87 (8.5%)	63 (7.5%)	24 (13.6%)	
**CNLC stage**				
Ia	396 (38.8%)	327 (38.7%)	69 (39.0%)	0.061
Ib	354 (34.7%)	297 (35.2%)	57 (32.2%)	
IIa	91 (8.9%)	78 (9.2%)	13 (7.3%)	
IIb	39 (3.8%)	36 (4.3%)	3 (1.7%)	
IIIa	141 (13.8%)	106 (12.6%)	35 (19.8%)	

AFP, alpha-fetoprotein; ALD, alcoholic liver disease; ALP, alkaline phosphatase; AJCC, American Joint Committee on Cancer; BCLC, Barcelona Clinic Liver Cancer; BDI, bile duct invasion; CNLC, China liver cancer; ES Grade, Edmondson-Steiner grade; GGT, gamma-glutamyltransferase; HCC, hepatocellular carcinoma; IQR, interquartile range; MaVI, macrovascular invasion; MELD, model for end-stage liver disease; MVI, microvascular invasion; NAFLD, non-alcoholic fatty liver disease; PS, performance status; TBil, total bilirubin.

### Overall Survival Analysis

**Figure 1:**
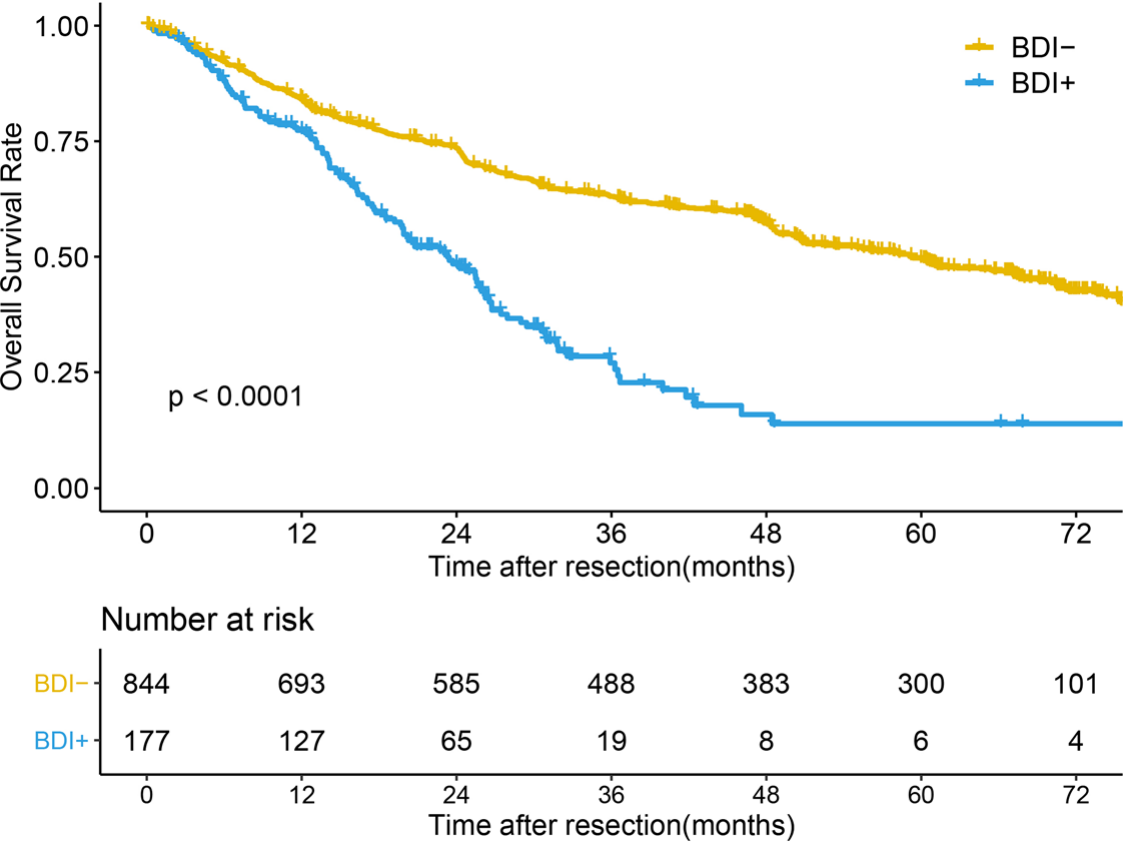
Overall survival of HCC without BDI and with BDI.

The median follow-up time of the whole cohort was 49.03 months (95% confidence interval [CI] 47.07-57.07 months). The median OS times (mOST) of patients without BDI and with BDI were 59.27 months (95%CI 50.7-67.17 months) and 23.3 months (95%CI 19.67-26.23 months), respectively (P<0.001) (**Figure 1**). The 1-, 3-, and 5-year OS rates of patients without BDI and with BDI were 84.2%, 63.0%, and 49.5% and 77.3%, 27.0%, and 13.9%, respectively. Multivariate analysis showed that BDI was an independent risk factor for OS (hazard ratio [HR]=2.047, 95% CI 1.619-2.589) (**Table 2**).

**Table 2:** Univariate and multivariate analysis of overall survival of HCC patients.

Characteristics	Univariate			Multivariate		
	HR	CI95%	*P*-value	HR	CI95%	*P*-value
Age, per year increase	0.998	0.991-1.006	0.674			
Sex, Male	1.055	0.831-1.34	0.659			
Underlying liver disease, Viral	1.177	0.981-1.411	0.079			
Cirrhosis, Yes	1.286	1.075-1.539	0.006	1.297	1.079-1.558	0.006
PS, per	2.628	2.238-3.085	<0.001	1.444	1.21-1.722	<0.001
Child-Pugh, B	2.256	1.743-2.921	<0.001			
MELD score, per	1.038	1.009-1.067	0.009			
Tumor Number, per	1.372	1.254-1.5	<0.001	1.093	0.991-1.206	0.076
Tumor Size, cm	1.113	1.092-1.133	<0.001	1.078	1.054-1.103	<0.001
ES grade, III/IV	1.915	1.411-2.599	<0.001	1.33	0.967-1.83	0.08
Capsule, Yes	0.738	0.56-0.973	0.031	0.722	0.541-0.964	0.027
Satellite, Yes	3.318	2.765-3.983	<0.001	1.55	1.242-1.935	<0.001
MVI, Yes	2.515	2.122-2.981	<0.001	1.437	1.182-1.747	<0.001
MaVI, Yes	4.757	3.842-5.889	<0.001	2.133	1.657-2.745	<0.001
Number of resected segments, per	1.397	1.284-1.519	<0.001	1.075	0.974-1.186	0.149
Major/minor liver resection, major	2.062	1.712-2.482	<0.001			
Preoperative adjuvant treatment, Yes	1.114	0.613-2.025	0.723			
Postoperative adjuvant treatment, Yes	0.913	0.77-1.083	0.295			
BDTT, Yes	2.361	1.908-2.923	<0.001	2.047	1.619-2.589	<0.001

BDI, bile duct invasion; ES Grade, Edmondson-Steiner grade; HCC, hepatocellular carcinoma; MaVI, macrovascular invasion; MELD, model for end-stage liver disease; MVI, microvascular invasion, PS, performance status.

**Figure 2:**
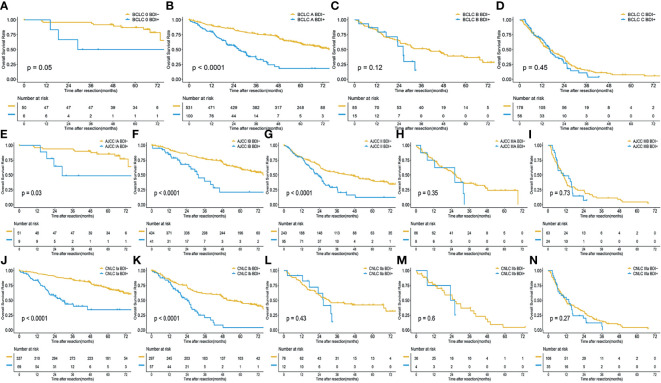
Overall survival of HCC without BDI and with BDI in each stage; **(A)** BCLC 0 stage; **(B)** BCLC A stage; **(C)** BCLC B stage; **(D)** BCLC C stage; **(E)** AJCC IA stage; **(F)** AJCC IB stage; **(G)** AJCC II stage; **(H)** AJCC IIIA stage; **(I)** AJCC IIIB stage; **(J)** CNLC Ia stage; **(K)** CNLC Ib stage; **(L)** CNLC II stage; **(M)** CNLC IIIa stage; **(N)** CNLC IIIb stage.

The respective mOST of the BDI- and BDI+ groups were 76.87 months (95%CI 76.87- Not Available [NA] months) and 28.98 months (95%CI 18.53-NA months) in BCLC stage 0 (P=0.05), 75.53 months (95%CI 70.37-NA months) and 26.73 months (95%CI 20.27-35.9 months) in BCLC stage A (P<0.001), 40.33 months (95%CI 27.43-67.6 months) and 26.2 months (95%CI 23.07-NA months) in BCLC stage B (P=0.12), and 16.2 months (95%CI 12.67-20.87 months) and 16.3 months (95%CI 13.17-22.73 months) in BCLC stage C (P=0.45) (**Figures 2A**–**D**).

The respective mOST of the BDI- and BDI+ groups were 76.87 months (95%CI 73.53-NA months) and 28.98 months (95%CI 23.43-NA months) in AJCC stage IA (P=0.03), 75.53 months (95%CI 70.07-NA months) and 31.8 months (95%CI 25.44-NA months) in AJCC stage IB (P<0.001), 44.97 months (95%CI 29.7-57.9 months) and 23.07 months (95%CI 19.67-26.53 months) in AJCC stage II (P<0.001), 26.93 months (95%CI 21.4-31.97 months) and 26.21 months (95%CI 12.6-NA months) in AJCC stage IIIA (P=0.35), 9.76 months (95%CI 7.57-12.63 months) and 10.23 months (95%CI 6.2-16.36 months) in AJCC stage IIIB (P=0.73) (**Figures 2E**–**I**).

The mOST of the BDI- and BDI+ groups were 80.83 months (95%CI 76.87-NA months) and 28.98 months (95%CI 20.2-NA months) in CNLC stage Ia (P<0.001), 52.6 months (95%CI 48.27-67.13 months) and 24.17 months (95%CI 19.67-30.97 months) in CNLC stage Ib (P<0.001), 30.47 months (95%CI 22.93-NA months)and 26.53 months (95%CI 17.07-NA months) in CNLC stage IIa (P=0.43), 26.2 months (95%CI 19.0-40.33 months) and 24.77 months (95%CI 7.57-NA months) in AJCC stage IIIA (P=0.6), 11.77 months (95%CI 9.7-17.7 months) and 13.96 months (95%CI 7.54-16.37 months) in AJCC stage IIIB (P=0.27) (**Figures 2J**–**N**).

### Construction of the Adjusted Staging Systems

**Figure 3:**
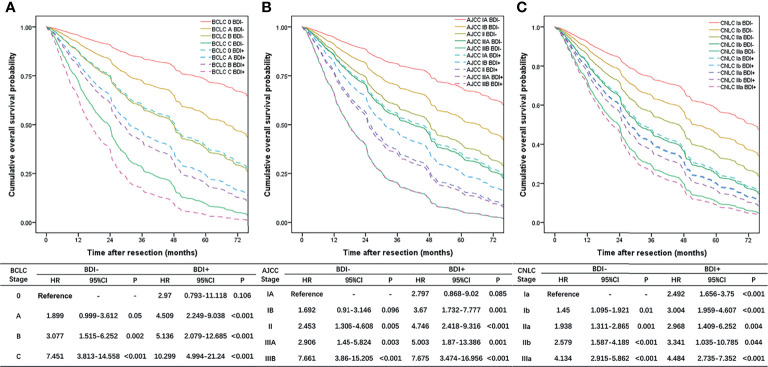
Overall survival curves stratified by different stages obtained from the multivariate Cox proportional hazard regression model adjusted for other covariates; **(A)** BCLC stage system; **(B)** eighth edition AJCC stage system; **(C)** CNLC stage system.

OS curves stratified by different stages obtained from the multivariate Cox proportional hazard regression model adjusted for other covariates are shown in **Figure 3**, and the multivariate Cox proportional hazard regression models are shown in **Supplementary Tables 1**–**3**. The prognosis of the BDI group in each stage was not better than that of the multiple tumors group, such as BCLC stage B (**Figure 3A**), AJCC stage IIIA (**Figure 3B**), and CNLC stage IIb (**Figure 3C**). The prognosis of BDI with macrovascular or major vascular invasion was not better than that of macrovascular or major vascular invasion, such as BCLC stage C (**Figure 3A**), AJCC stage IIIB (**Figure 3B**), and CNLC stage IIIa (**Figure 3C**).

**Figure 4:**
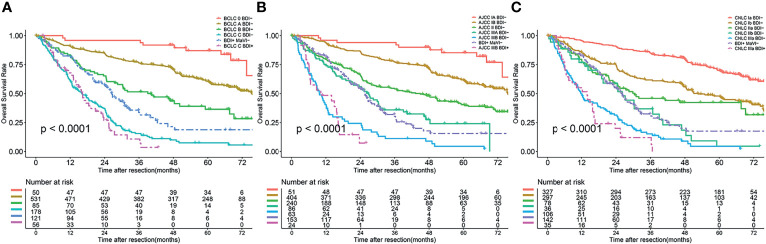
Overall survival curves of patients after BDI regrouping in different stage systems; **(A)** BCLC stage system; **(B)** eighth edition AJCC stage system; **(C)** CNLC stage system.

The BDI+ group was restaged according to the HR of each stage. BCLC stage 0/A/B BDI+ was integrated into BDI+MaVI- and classified as BCLC stage B, and BCLC stage C BDI+ was classified as BCLC stage C (**Figure 4A**). The definition of the adjusted BCLC stage B was multinodular or bile duct invasion, preserved liver function, and PS 0, with the rest of the BCLC stage remaining unchanged.

AJCC stage IA/IB/II/IIIA BDI+ was integrated into BDI+MaVI- and classified as AJCC stage IIIA, and AJCC stage IIIB BDI+ was classified as AJCC stage IIIB (**Figure 4B**). The definition of the adjusted AJCC stage T3 was multiple tumors, at least one of which was measuring > 5 cm, or bile duct invasion, with the rest of the AJCC stage remaining unchanged.

CNLC stage Ia/Ib/IIa/IIb BDI+ was integrated into BDI+MaVI- and classified as CNLC stage IIb, and CNLC stage IIIa BDI+ was classified as CNLC stage IIIa (**Figure 4C**). The definition of the adjusted CNLC stage IIb was PS 0–2, liver function Child–Pugh grade A/B, ≥ 4 tumors or bile duct invasion, any tumor diameter, and no vascular invasion and extrahepatic metastasis, with the rest of the CNLC stage remaining unchanged.

The definitions of adjusted staging systems are shown in **Supplementary Table 4**.

### Predictive Performance of the Adjusted Staging Systems

**Figure 5:**
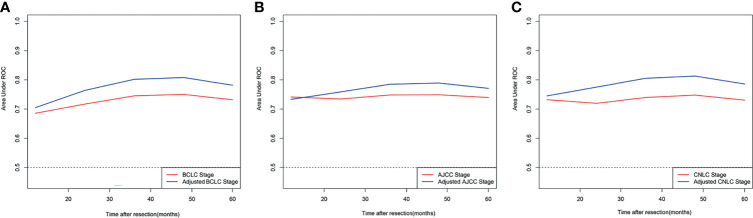
Comparison of time-dependent receiver operating characteristic curve of different adjusted stage system and corresponding current stage system; **(A)** BCLC stage system; **(B)** eighth edition AJCC stage system; **(C)** CNLC stage system.

A comparison of the C-index of the current staging systems and the corresponding adjusted staging systems is shown in **Table 3**. The C-indexes of the BCLC staging system and adjusted BCLC staging system were 0.666 and 0.695, respectively (P<0.001). The C-indexes of the AJCC staging system and adjusted AJCC staging system were 0.676 and 0.688, respectively (P=0.049). The C-indexes of the CNLC staging system and adjusted CNLC staging system were 0.676 and 0.703, respectively (P=0.002). All tdAUCs of the adjusted staging systems were higher than those of the current staging systems except for the 1-year tdAUCs of the AJCC and CNLC stages (all P<0.05); the 1-year tdAUC values of the AJCC and CNLC stages were not significantly different when compared with those of the adjusted staging systems (all P>0.05) (**Table 4** and **Figures 5A**–**C**).

**Table 3:** C-index comparison of different stages.

Stage System	C-Index		P
	Current stage	Adjusted stage	
BCLC *vs.* Adjusted BCLC	0.666	0.695	<0.001
AJCC *vs.* Adjusted AJCC	0.676	0.688	0.049
CNLC *vs.* Adjusted CNLC	0.676	0.703	0.002

AJCC, American Joint Committee on Cancer; BCLC, Barcelona Clinic Liver Cancer; CNLC, China liver cancer.

**Table 4:** Time-dependent AUC comparison of different stages.

tdAUC	BCLC	Adjusted BCLC	P	AJCC	Adjusted AJCC	P	CNLC	Adjusted CNLC	P
1-year	0.686	0.705	0.041	0.741	0.733	0.419	0.732	0.745	0.363
2-year	0.723	0.763	<0.001	0.734	0.756	0.012	0.725	0.772	<0.001
3-year	0.748	0.801	<0.001	0.746	0.783	<0.001	0.743	0.804	<0.001
4-year	0.752	0.808	<0.001	0.748	0.788	<0.001	0.751	0.812	<0.001
5-year	0.733	0.781	<0.001	0.738	0.77	<0.001	0.733	0.785	<0.001

AJCC, American Joint Committee on Cancer; BCLC, Barcelona Clinic Liver Cancer; CNLC, China liver cancer.

## Discussion

Compared with patients without BDI, those with BDI often had a higher Child–Pugh grade, TBil level, AFP level, GGT level, ALP level, and higher incidence rates of MVI and MaVI. Studies have indicated that BDI has more aggressive characteristics with a higher positive expression rate of the liver stem cell markers C-Kit, CK-19, CD90, CD133, and EpCAM (14, 18). However, the impact of BDI (HR=2.361, 95%CI 1.908-2.923) on OS seemed less prominent than macrovascular invasion (HR=4.757, 95%CI 3.842-5.889), which is consistent with that in previous reports (8, 13, 24).

Should BDI be considered a component of the staging systems? Two previous studies have attempted to answer this question. A Korean–Japanese multicenter study (8) included 257 patients with BDI from 32 centers. The results suggested that BDI is not an independent risk factor for survival, and the seventh AJCC stage can well distinguish these patients. However, only patients with BDI were included in this study, and no patients without BDI were included as controls. Another single-center study (25) analyzed 19 patients with BDI and 600 HCC patients without BDI, the results showed that HCC with BDI should be classified as BCLC stage B. However, the sample size of BDI was small, the BDI patients were not grouped when analyzed, and no significant differences in receiver operating characteristic curves were found between the original BCLC and modified BCLC systems at 1, 3, and 5 years (P>0.05). The two aforementioned studies have some limitations; thus, we explored these limitations further.

In this study, we retrospectively analyzed a large cohort of data of patients with HCC from nine large hepatobiliary medical centers. The results showed that BDI was an independent risk factor for OS in patients with HCC. The results of the BCLC stage, AJCC stage, and CNLC stage showed that the OS of HCC patients with BDI in the early stage was significantly worse than that of those without BDI in the same stage whereas the difference was no significant in intermediate stage or advanced stage. This indicates that the prognosis of BDI patients is heterogeneous. For the convenience of clinical use, considered both prognosis and treatment options, we divided patients with BDI into BDI without MaVI and BDI with MaVI, constructed adjusted staging systems on the basis of the current staging systems, not only significantly improved the prediction performance but also applies to treatment selection.

Though BDI is a sign of poor prognosis, BDI is not a contraindication for surgery. When the tumor invades the vascular system, the tumor cells may metastasize *via* the blood stream; thus, the patient may not achieve complete resection. In contrast to macrovascular invasion, tumor cells enter the intestinal tract with bile after bile duct invasion. This may be because of the digestive fluid, and metastasis rarely occurs in the intestinal tract; therefore, surgical resection of the tumor and BDI may obtain satisfactory results. Several studies have compared the prognosis of BDI patients following different treatment methods. Results indicated that HCC patients with BDI who underwent resection achieved the best prognosis with a median survival time of 11.5–47 months (9, 26–28), patients who received transcatheter chemoembolization or systemic chemotherapy had a median survival time of 6–11 months (26–28), and patients who received conservative management had a median survival time of only 1.6–4.3 months (26–28). And most researchers suggest that patients with BDI should receive radical surgery if conditions permit (6, 8–11, 16, 24). Therefore, in terms of guidance for treatment selection, the adjusted staging systems showed no change to the current staging systems.

There are some limitations to this study. First, there was unavoidable selection bias in this retrospective analysis. Second, due to the lack of data, other tumor factors such as gene mutation cannot be included in multivariate analysis, which limits the predictive ability of the model. Third, patients with BDI who received non-surgical treatment were not included. Last, all patients were from China, and most patients had a background of hepatitis B virus infection, further external validation is needed.

## Conclusion

According to the BDI status, we adjusted the current staging systems to help clinicians make a more accurate prognostic evaluation.

## Data Availability Statement

The raw data supporting the conclusions of this article will be made available by the authors, without undue reservation.

## Author Contributions

Study concept and design: QH, YC, KL, JL, and YYZ. Acquisition, analysis, or interpretation of data: All authors. Statistical analysis: QH and KL. Drafting of the manuscript: QH, YC, KL, JL, and YYZ. Critical revision of the manuscript for important intellectual content: All authors. Administrative, technical, or material support: YC, CS, SZ, JC, YW, YMZ, WZ, JL, and YYZ. All authors contributed to the article and approved the submitted version.

## Funding

This study was supported by Science and Technology project of Fuzhou (Grant number: 2020-WS-127), Key Clinical Specialty Discipline Construction Program of Fuzhou, Fujian, P.R.C (Grant number: 201912002) and Fujian Provincial Medical Center of Hepatobiliary.

## Conflict of Interest

The authors declare that the research was conducted in the absence of any commercial or financial relationships that could be construed as a potential conflict of interest.

## Publisher’s Note

All claims expressed in this article are solely those of the authors and do not necessarily represent those of their affiliated organizations, or those of the publisher, the editors and the reviewers. Any product that may be evaluated in this article, or claim that may be made by its manufacturer, is not guaranteed or endorsed by the publisher.
