# Supplementary figures and images for: Redefining Hepatocellular Carcinoma Staging Systems Based on the Bile Duct Invasion Status: A Multicenter Study
Source: Front Oncol. 2021 Oct 14;11:673285. doi: 10.3389/fonc.2021.673285 (PMC8551376; doi:10.3389/fonc.2021.673285)

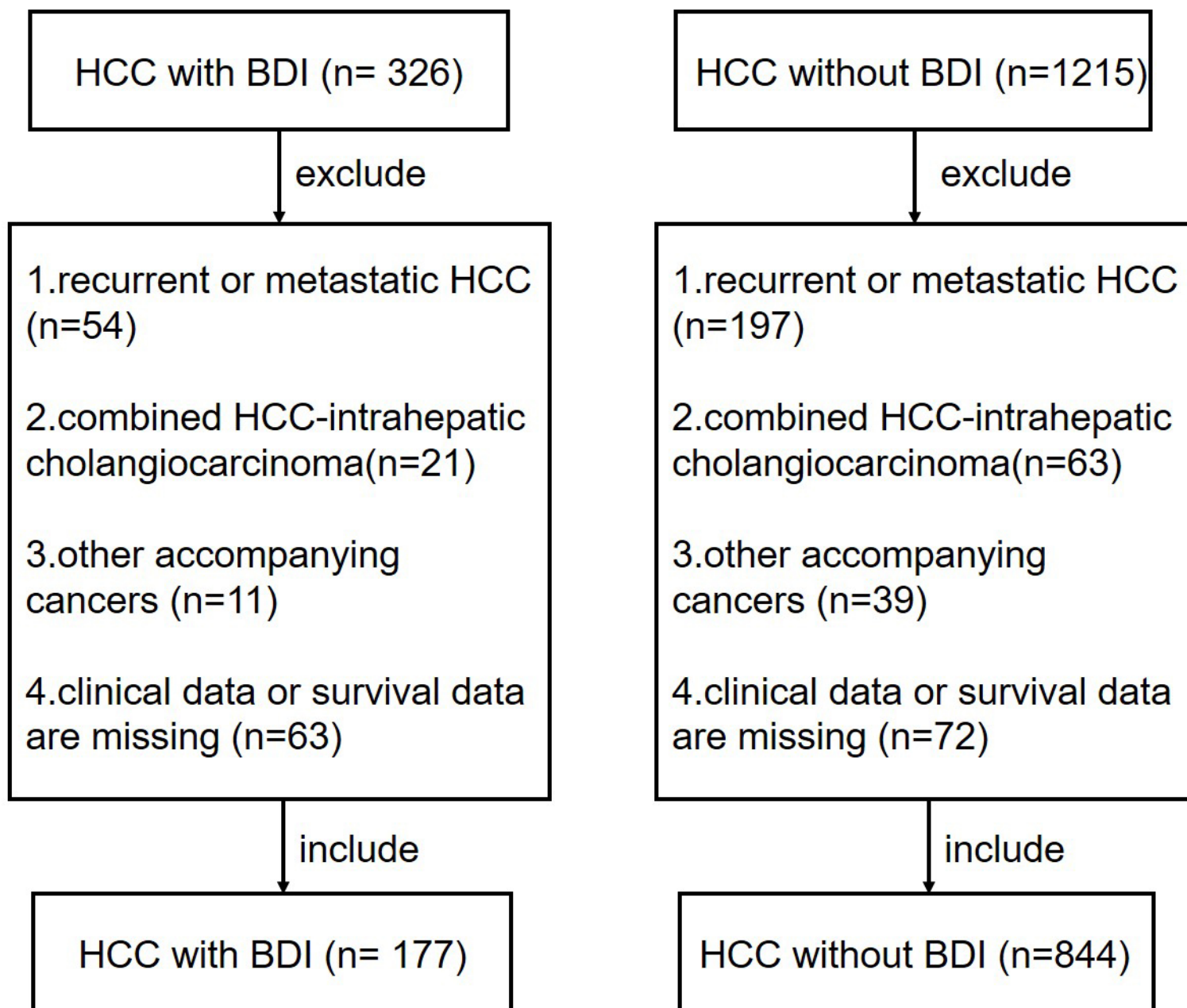

Supplement: Supplementary file 1 [file Image_1.pdf]
