# Supplementary material for: Redefining Hepatocellular Carcinoma Staging Systems Based on the Bile Duct Invasion Status: A Multicenter Study
Source: Front Oncol. 2021 Oct 14;11:673285. doi: 10.3389/fonc.2021.673285 (PMC8551376; doi:10.3389/fonc.2021.673285)
Supplement: Supplementary file 2 [file Table_1.docx]

**Supplementary Table 1** Univariate and multivariate Cox proportional hazard regression model for development of BCLC staging system

| **Characteristics** | **Univariate** | | |  | **Multivariate** | | |
| --- | --- | --- | --- | --- | --- | --- | --- |
|  | **HR** | **CI95%** | ***P*-value** |  | **HR** | **CI95%** | ***P*-value** |
| Age (years) | 0.998 | 0.991-1.006 | 0.674 |  |  |  |  |
| Sex, Male | 1.055 | 0.831-1.34 | 0.659 |  |  |  |  |
| Underlying liver disease, Viral | 1.222 | 0.991-1.506 | 0.061 |  |  |  |  |
| Cirrhosis, Yes | 1.286 | 1.075-1.539 | 0.006 |  | 1.296 | 1.077-1.56 | 0.006 |
| PS, per | 2.628 | 2.238-3.085 | <0.001 |  |  |  |  |
| Child-Pugh, B | 2.256 | 1.743-2.921 | <0.001 |  |  |  |  |
| TBil (umol/L) | 1.003 | 1.002-1.004 | <0.001 |  |  |  |  |
| ALP (U/L) | 1.001 | 1.001-1.001 | <0.001 |  |  |  |  |
| GGT (U/L) | 1.002 | 1.001-1.002 | <0.001 |  | 1.001 | 1.000-1.001 | 0.004 |
| AFP (ng/mL) | 1.000 | 1.000-1.000 | <0.001 |  |  |  |  |
| Tumor number, Multiple | 1.372 | 1.254-1.5 | <0.001 |  |  |  |  |
| Tumor size (cm) | 1.113 | 1.092-1.133 | <0.001 |  | 1.083 | 1.06-1.106 | <0.001 |
| ES grade, III/IV | 1.915 | 1.411-2.599 | <0.001 |  |  |  |  |
| Capsule, Yes | 0.738 | 0.56-0.973 | 0.031 |  | 0.682 | 0.513-0.906 | 0.008 |
| Satellite nodules, Yes | 3.318 | 2.765-3.983 | <0.001 |  | 1.231 | 1.005-1.508 | 0.045 |
| Microvascular invasion, Yes | 2.515 | 2.122-2.981 | <0.001 |  | 1.394 | 1.14-1.704 | <0.001 |
| Macrovascular invasion, Yes | 4.757 | 3.842-5.889 | <0.001 |  |  |  |  |
| Preoperative treatment, Yes | 1.114 | 0.613-2.025 | 0.723 |  |  |  |  |
| Postoperative adjuvant treatment, Yes | 0.913 | 0.77-1.083 | 0.295 |  |  |  |  |
| BCLC Stage (Ref=BCLC 0 BDI-) | Reference |  | <0.001 |  | Reference |  | <0.001 |
| BCLC A BDI- | 2.522 | 1.339-4.753 | 0.004 |  | 1.899 | 0.999-3.612 | 0.05 |
| BCLC B BDI- | 5.454 | 2.762-10.77 | <0.001 |  | 3.077 | 1.515-6.252 | 0.002 |
| BCLC C BDI- | 15.112 | 7.913-28.858 | <0.001 |  | 7.451 | 3.813-14.558 | <0.001 |
| BCLC 0 BDI+ | 3.702 | 1.009-13.583 | 0.048 |  | 2.97 | 0.793-11.118 | 0.106 |
| BCLC A BDI+ | 8.239 | 4.198-16.171 | <0.001 |  | 4.509 | 2.249-9.038 | <0.001 |
| BCLC B BDI+ | 10.478 | 8.622-25.345 | <0.001 |  | 5.136 | 2.079-12.685 | <0.001 |
| BCLC C BDI+ | 17.359 | 4.753-34.949 | <0.001 |  | 10.299 | 4.994-21.24 | <0.001 |

Abbreviations: AFP, alpha-fetoprotein; ALP, alkaline phosphatase; BCLC, Barcelona Clinic Liver Cancer; BDI, bile duct invasion; ES Grade, Edmondson-Steiner grade; GGT, gamma-glutamyltransferase; PS, performance status; TBil, total bilirubin.

**Supplementary Table 2** Univariate and multivariate Cox proportional hazard regression model for development of 8^th^ AJCC staging system

|  | **Univariate** | | |  | **Multivariate** | | |
| --- | --- | --- | --- | --- | --- | --- | --- |
| **Characteristics** | **HR** | **CI95%** | ***P*-value** |  | **HR** | **CI95%** | ***P*-value** |
| Age (years) | 0.998 | 0.991-1.006 | 0.674 |  |  |  |  |
| Sex, Male | 1.055 | 0.831-1.34 | 0.659 |  |  |  |  |
| Underlying liver disease, Viral | 1.222 | 0.991-1.506 | 0.061 |  |  |  |  |
| Cirrhosis, Yes | 1.286 | 1.075-1.539 | 0.006 |  | 1.342 | 1.115-1.615 | 0.002 |
| PS, per | 2.628 | 2.238-3.085 | <0.001 |  | 1.776 | 1.5-2.102 | <0.001 |
| Child-Pugh, B | 2.256 | 1.743-2.921 | <0.001 |  |  |  |  |
| TBil (umol/L) | 1.003 | 1.002-1.004 | <0.001 |  |  |  |  |
| ALP (U/L) | 1.001 | 1.001-1.001 | <0.001 |  |  |  |  |
| GGT (U/L) | 1.002 | 1.001-1.002 | <0.001 |  | 1.001 | 1.000-1.001 | 0.002 |
| AFP (ng/mL) | 1.000 | 1.000-1.000 | <0.001 |  |  |  |  |
| Tumor number, Multiple | 1.372 | 1.254-1.5 | <0.001 |  |  |  |  |
| Tumor size (cm) | 1.113 | 1.092-1.133 | <0.001 |  | 1.085 | 1.062-1.109 | <0.001 |
| ES grade, III/IV | 1.915 | 1.411-2.599 | <0.001 |  |  |  |  |
| Capsule, Yes | 0.738 | 0.56-0.973 | 0.031 |  | 0.678 | 0.511-0.9 | 0.007 |
| Satellite nodules, Yes | 3.318 | 2.765-3.983 | <0.001 |  | 1.299 | 1.053-1.601 | 0.014 |
| Microvascular invasion, Yes | 2.515 | 2.122-2.981 | <0.001 |  |  |  |  |
| Major vascular invasion, Yes | 5.188 | 4.033-6.673 | <0.001 |  |  |  |  |
| Preoperative treatment, Yes | 1.114 | 0.613-2.025 | 0.723 |  |  |  |  |
| Postoperative adjuvant treatment, Yes | 0.913 | 0.77-1.083 | 0.295 |  |  |  |  |
| 8th AJCC Stage (Ref=AJCC IA BDI-) | Reference |  | <0.001 |  | Reference |  | <0.001 |
| AJCC IB BDI- | 2.226 | 1.209-4.096 | 0.01 |  | 1.692 | 0.91-3.146 | 0.096 |
| AJCC II BDI- | 4.178 | 2.26-7.723 | <0.001 |  | 2.453 | 1.306-4.608 | 0.005 |
| AJCC IIIA BDI- | 7.331 | 3.828-14.039 | <0.001 |  | 2.906 | 1.45-5.824 | 0.003 |
| AJCC IIIB BDI- | 18.07 | 9.419-34.665 | <0.001 |  | 7.661 | 3.86-15.205 | <0.001 |
| AJCC IA BDI+ | 3.435 | 1.086-10.864 | 0.036 |  | 2.797 | 0.868-9.02 | 0.085 |
| AJCC IB BDI+ | 5.366 | 2.561-11.241 | <0.001 |  | 3.67 | 1.732-7.777 | 0.001 |
| AJCC II BDI+ | 9.033 | 4.737-17.224 | <0.001 |  | 4.746 | 2.418-9.316 | <0.001 |
| AJCC IIIA BDI+ | 10.873 | 4.196-28.178 | <0.001 |  | 5.003 | 1.87-13.386 | 0.001 |
| AJCC IIIB BDI+ | 22.492 | 10.654-47.486 | <0.001 |  | 7.675 | 3.474-16.956 | <0.001 |

Abbreviations: AFP, alpha-fetoprotein; ALP, alkaline phosphatase; AJCC, American Joint Committee on Cancer; BDI, bile duct invasion; ES Grade, Edmondson-Steiner grade; GGT, gamma-glutamyltransferase; PS, performance status; TBil, total bilirubin.

**Supplementary Table 3** Univariate and multivariate Cox proportional hazard regression model for development of CNLC staging system

| **Characteristics** | **Univariate** | | |  | **Multivariate** | | |
| --- | --- | --- | --- | --- | --- | --- | --- |
|  | **HR** | **CI95%** | ***P*-value** |  | **HR** | **CI95%** | ***P*-value** |
| Age (years) | 0.998 | 0.991-1.006 | 0.674 |  |  |  |  |
| Sex, Male | 1.055 | 0.831-1.34 | 0.659 |  |  |  |  |
| Underlying liver disease, Viral | 1.222 | 0.991-1.506 | 0.061 |  |  |  |  |
| Cirrhosis, Yes | 1.286 | 1.075-1.539 | 0.006 |  | 1.306 | 1.084-1.574 | 0.005 |
| PS, per | 2.628 | 2.238-3.085 | <0.001 |  | 1.525 | 1.279-1.819 | <0.001 |
| Child-Pugh, B | 2.256 | 1.743-2.921 | <0.001 |  |  |  |  |
| Tbil (umol/L) | 1.003 | 1.002-1.004 | <0.001 |  |  |  |  |
| ALP (U/L) | 1.001 | 1.001-1.001 | <0.001 |  |  |  |  |
| GGT (U/L) | 1.002 | 1.001-1.002 | <0.001 |  | 1.001 | 1-1.001 | 0.004 |
| AFP (ng/mL) | 1.000 | 1.000-1.000 | <0.001 |  |  |  |  |
| Tumor number, Multiple | 1.372 | 1.254-1.5 | <0.001 |  |  |  |  |
| Tumor size (cm) | 1.113 | 1.092-1.133 | <0.001 |  | 1.072 | 1.046-1.098 | <0.001 |
| ES grade, III/IV | 1.915 | 1.411-2.599 | <0.001 |  |  |  |  |
| Capsule, Yes | 0.738 | 0.56-0.973 | 0.031 |  | 0.718 | 0.541-0.953 | 0.022 |
| Satellite nodules, Yes | 3.318 | 2.765-3.983 | <0.001 |  | 1.273 | 1.035-1.566 | 0.023 |
| Microvascular invasion, Yes | 2.515 | 2.122-2.981 | <0.001 |  | 1.384 | 1.125-1.703 | 0.002 |
| Macrovascular invasion, Yes | 4.757 | 3.842-5.889 | <0.001 |  |  |  |  |
| Preoperative treatment, Yes | 1.114 | 0.613-2.025 | 0.723 |  |  |  |  |
| Postoperative adjuvant treatment, Yes | 0.913 | 0.77-1.083 | 0.295 |  |  |  |  |
| CNLC Stage (Ref=CNLC Ia BDI-) | Reference |  | <0.001 |  | Reference |  | <0.001 |
| CNLC Ib BDI- | 2.175 | 1.704-2.775 | <0.001 |  | 1.45 | 1.095-1.921 | 0.01 |
| CNLC IIa BDI- | 3.141 | 2.214-4.456 | <0.001 |  | 1.938 | 1.311-2.865 | 0.001 |
| CNLC IIb BDI- | 5.498 | 3.566-8.476 | <0.001 |  | 2.579 | 1.587-4.189 | <0.001 |
| CNLC IIIa BDI- | 9.614 | 7.187-12.863 | <0.001 |  | 4.134 | 2.915-5.862 | <0.001 |
| CNLC Ia BDI+ | 3.436 | 2.331-5.064 | <0.001 |  | 2.492 | 1.656-3.75 | <0.001 |
| CNLC Ib BDI+ | 5.99 | 4.127-8.694 | <0.001 |  | 3.004 | 1.959-4.607 | <0.001 |
| CNLC IIa BDI+ | 5.321 | 2.579-10.979 | <0.001 |  | 2.968 | 1.409-6.252 | 0.004 |
| CNLC IIb BDI+ | 6.441 | 2.035-20.386 | 0.002 |  | 3.341 | 1.035-10.785 | 0.044 |
| CNLC IIIa BDI+ | 12.361 | 8.08-18.911 | <0.001 |  | 4.484 | 2.735-7.352 | <0.001 |

Abbreviations: AFP, alpha-fetoprotein; ALP, alkaline phosphatase; BDI, bile duct invasion; CNLC, China liver cancer; ES Grade, Edmondson-Steiner grade; GGT, gamma-glutamyltransferase; PS, performance status; TBil, total bilirubin.

**Supplementary Table 4** Definitions of adjusted staging systems.

| **BCLC** | **BCLC stage 0** | **BCLC stage A** | **BCLC stage B** | **BCLC stage C** | **BCLC stage D** |  |  |
| --- | --- | --- | --- | --- | --- | --- | --- |
| Definition | single tumour <2 cm in diameter without vascular invasion/satellites, preserved liver function, PS 0. | single tumours >2 cm or three nodules <3 cm in diameter, preserved liver function, PS 0. | multinodular or bile duct invasion, preserved liver function, PS 0. | portal invasion/extrahepatic spread, preserved liver function, PS 1-2. | Not transplantable HCC, end-stage liver function, PS 3-4. |  |  |
| **AJCC*** | **TX** | **T0** | **T1a** | **T1b** | **T2** | **T3** | **T4** |
| Definition | Primary tumor cannot be assessed | No evidence of primary tumor | Solitary tumor <2 cm | Solitary tum or >2 cm without vascular invasion | Solitary tum or >2 cm with vascular invasion, or multiple tumors, none >5 cm | Multiple tumors, at least one of which is >5 cm, or bile duct invasion | Single tumor or multiple tumors of any size involving a major branch of the portal vein or hepatic vein, or tumor(s) with direct invasion of adjacent organs other than the gallbladder or with perforation of visceral peritoneum |
| CNLC | **CNLC Stage Ia** | **CNLC Stage Ib** | **CNLC Stage IIa** | **CNLC Stage IIb** | **CNLC Stage IIIa** | **CNLC Stage IIIb** | **CNLC Stage IV** |
| Definition | a performance status (PS) score of 0 to 2, Child-Pugh A/B liver function, a solitary tumor with a diameter of ≤5 cm, and absence of vascular invasion or extrahepatic metastasis. | a PS score of 0 to 2, Child-Pugh A/B liver function, a solitary tumor with a diameter of >5 cm, or 2 to 3 tumors with a maximum diameter ≤3 cm, and absence of vascular invasion or extrahepatic metastasis. | a PS score of 0 to 2, Child-Pugh A/B liver function, 2 to 3 tumors with a maximum diameter >3 cm, and absence of vascular invasion or extrahepatic metastasis. | a PS score of 0 to 2, Child-Pugh A/B liver function, bile duct invasion or ≥4 tumors regardless of tumor diameter, and absence of vascular invasion or extrahepatic metastasis. | a PS score of 0 to 2, Child-Pugh A/B liver function, regardless of tumor status, and presence of vascular invasion but absence of extrahepatic metastasis. | a PS score of 0 to 2, Child-Pugh A/B liver function, regardless of tumor status and vascular invasion, and presence of extrahepatic metastasis. | a PS score of 3 to 4, Child-Pugh C liver function, regardless of tumor status, vascular invasion, and extrahepatic metastasis. |

The highlight is the content of the change.

*T stage was adjusted, N stage and M stage was unchanged, stages are still judged according to the combination of TNM.
